# Information and Knowledge Diffusion Dynamics in Complex Networks with Independent Spreaders

**DOI:** 10.3390/e27030234

**Published:** 2025-02-24

**Authors:** Yan Zhuang, Weihua Li, Yang Liu

**Affiliations:** 1 School of Economics and Management, Beihang University, Beijing 100191, China; zhuangyan@buaa.edu.cn; 2 School of Software, Beihang University, Beijing 100191, China; 3 LMIB, NLSDE, BDBC, and School of Artificial Intelligence, Beihang University, Beijing 100191, China; 4 Hangzhou International Innovation Institute, Beihang University, Hangzhou 311115, China; 5 Department of Strategic and Advanced Interdisciplinary Research, Pengcheng Laboratory, Shenzhen 518055, China; 6 Zhongguancun Laboratory, Beijing 100080, China; 7 Qianyuan Laboratory, Hangzhou 310024, China; 8 Beijing Advanced Innovation Center for Future Blockchain and Privacy Computing, Beihang University, Beijing 100191, China; 9 Communist Youth League Committee, Beihang University, Beijing 100191, China

**Keywords:** complex networks, information diffusion, innovation, knowledge diffusion, epidemic models, threshold

## Abstract

Information and knowledge diffusion are important dynamical processes in complex social systems, in which the underlying topology of interactions among individuals is often modeled as networks. Recent studies have examined various information diffusion scenarios primarily focusing on the dynamics within one network; yet, relatively little scholarly attention has been paid to possible interactions among individuals beyond the focal network. Here, in this study, we account for this phenomenon by modeling the information diffusion dynamics with the involvement of independent spreaders in a susceptible–exposed–infectious–recovered contagion process. Independent spreaders receive information using latent information transmission pathways without following the links in the focal network and can spread the information to remote areas of the network not well connected to the major components. We derive the mathematics of the critical epidemic thresholds on homogeneous and heterogeneous networks as a function of the infectious rate, exposure rate, recovery rate and the activeness of independent spreaders. We present simulation results on Small World and Scale-Free complex networks, and real-world social networks of Facebook artists and physicist collaborations. The result shows that the extent to which information or knowledge can spread might be more extensive than we can explain in terms of link contagion only. In addition, these results also help to explain how the activeness of independent spreaders can affect the diffusion process of information and knowledge in complex networks, which may have implications for studies exploring other dynamical processes.

## 1. Introduction

Information diffusion and knowledge sharing on complex social networks are important dynamical processes in real-world spreading phenomena, which have attracted broad scholarly attention, such as computer scientists, physicists, epidemiologists, and social scientists. Such diffusion dynamics appear in many empirical social networks, such as online social platforms like Facebook, Twitter, and Instagram [1,2,3,4], international alliance networks [5], contact networks of humans and/or animals [6,7,8], collaboration networks of scientists [9,10,11], and many more. For example, users on online social platforms share news, events, and ideas with their friends, and the information they spread can go viral to reach a large audience with whom they have no direct connections. Animals such as schooling fish can form groups to communicate with neighboring individuals, which allows them to predate, move, and avoid obstacles and enemies collectively. Researchers form collaboration networks to collectively solve complex scientific problems and share knowledge and expertise. The knowledge-sharing process also exists among companies, research institutions, universities, and other entities. These examples from diverse domains demonstrate the breadth and importance of information diffusion in complex social networks.

Sharing information in real-world scenarios is a complicated process that involves human thinking and activities [12,13,14,15,16,17,18]. Typically, there is often an interval for individuals between learning the information or knowledge and the subsequent activity of spreading it [19,20,21,22,23]. This interval between learning and sharing information or knowledge is more profound among research institutions or universities. We adopted the susceptible (S)–exposed (E)–infected (I)–recovered (R) epidemic modeling scheme for our information diffusion model [24,25]. Compared to other popular epidemic or information diffusion models, the exposed state of nodes accounts for the period that nodes receiving the information or knowledge hesitate and prepare to share the information with other individuals. This modeling framework can more accurately emulate real-world spreading processes than other SIS or SIR models.

We then accounted for the fact that many individuals acquire the information or knowledge from other channels rather than through the normal link contagion of neighboring spreaders. For example, one can obtain information from Twitter and then post it on Facebook. We call these individuals independent spreaders, and they do not receive information from other spreaders but become spreaders themselves with a certain probability, which we call the activeness of independent spreaders [26,27,28]. We then compared the independent spreaders with other potential mechanisms proposed in recent research that may also insert exogenous influence into the system, such as external seeding and hybrid diffusion in complex networks [29,30,31,32,33,34,35].

We are concerned with the evolution of the number and location of infected nodes of the network as a function of time and aim to understand the property of diffusion dynamics in the equilibrium and long-time steady state, the existence of a non-zero density of infected nodes, and the presence and absence of a global information outbreak. This model can also be applied to the knowledge-sharing dynamics of scholars in universities and research institutions, providing new theoretical and numerical evidence for assessing the innovation processes in universities.

## 2. Results

In this study, we propose an SEIR information diffusion model in complex networks with the participation of independent spreaders. At the beginning of the diffusion process, infected nodes that know the information or knowledge can transmit the information to the neighboring susceptible nodes with infection rate β. The infected nodes become exposed, which represents the state when individuals have learned the information or knowledge, but have not decided or been ready to share it with other individuals in the network. Exposed nodes will enter the infected state with rate α, and infected nodes can transmit the information to other connected susceptible nodes. Infected nodes can recover with a probability μ and will no longer be active in the information diffusion process.

**Figure 1:**
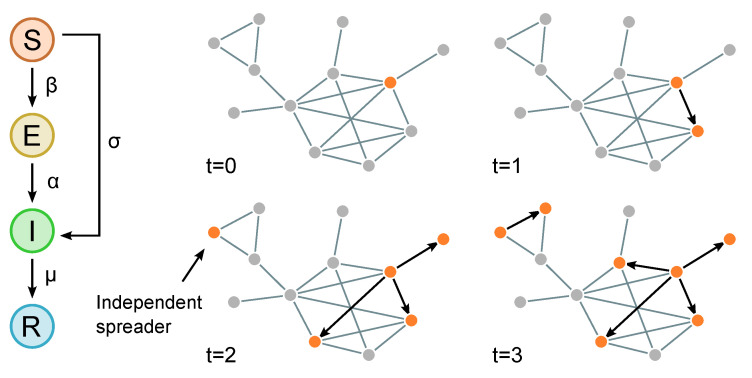
Illustrative figure of the SEIR diffusion dynamics with independent spreaders. In the left panel, we plot the key model parameters and how nodes from four compartments move to other states in the diffusion process. In the right panel, we exhibit a typical diffusion process with an independent spreader, which appears in t=3 in an area less connected to other parts of the network.

Independent spreaders are defined as nodes infected, not via link contagion, but through the links of complex networks. We assume that each susceptible node can become infected with a rate proportional to σ, which indicates the activeness of independent spreaders and the infection rate in the current system. In real-world scenarios, independent spreaders can be those individuals that acquire information or knowledge from external sources, such as other online social platforms or media. The rate of σ represents the tendency or ability of this external influence to cause information sharing in the focal social network, and a high σ indicates that the external environment has a greater impact on the information sharing or that the specific information has global influence beyond this network. We present an illustrative plot of the role of independent spreaders in Figure 1.

We analyzed the diffusion outbreak time scale in the initial stage and the long-term outbreak size of the SEIR information diffusion dynamic systems in homogeneous networks and heterogeneous networks and provided analytical solutions for these crucial quantities of the system. We also ran extensive computational simulations on synthetic homogeneous and heterogeneous networks, as well as two large-scale real-world social networks, one being the Facebook artist network and the other being the physicist collaboration network.

### 2.1. Information Diffusion on Homogeneous Networks

We first discuss our information diffusion model with independent spreaders in homogeneous networks. In this setting, the underlying complex social network was regarded as homogeneous in its topology, and we assumed that each node has the same degree equal to the average degree 〈k〉 of the network. The nodes belong to one of the four compartments during any time of the diffusion process, and the number of susceptible, exposed, infected, and recovered nodes at time *t* are indicated as S(t), E(t), I(t), and R(t), respectively. Equivalently, the corresponding density of susceptible, exposed, infected, and recovered nodes is s(t)=S(t)/N, e(t)=E(t)/N, i(t)=I(t)/N, and r(t)=R(t)/N, respectively, where *N* is the total number of nodes in the network.

In the homogeneous assumption, the force of information diffusion is proportional to the average number of contacts with infected nodes. Since each infected node attempts to transfer the information to a connected susceptible node with probability βdt, where β is the spreading rate of the information, a susceptible node with degree *k* will have on average ki(t) connections with infected nodes, yielding at the leading order of dt→0 an infection acquisition probability of $1-{\left(1-\beta dt\right)}^{ki(t)}≃\beta ki\left(t\right)dt$. The nodes receiving the information will become exposed, entering a phase where they will become infected to share the information proportional to αdt. The exposed state represents the situation where individuals have acquired the information or knowledge but hesitate to spread it. In academic collaborations, this process also accounts for the fact that the diffusion of the latest scientific knowledge takes time for researchers to digest and adopt.

The above considerations are typical for an epidemic model; yet, in real-world social networks, information can be accessed and spread via multiple pathways. For instance, users can learn the information from one platform and share it on another. Researchers may also independently develop similar scientific knowledge without having to learn from collaborators. To account for this phenomenon, we introduced independent spreaders, which denote the set of nodes that become infected without infection from other nodes via connected links in the network. The independent spreaders randomly appear in the system at a rate of σ. With the participation of an independent spreader, the dynamics of susceptible nodes can be modified by a term −σs(t)i(t) as the rate to become infected, and similarly the evolution of infected nodes will also have a new term σs(t)i(t).

The SEIR information diffusion model is specified in the following equations.

(1) $$\begin{array}{cc} \frac{ds(t)}{dt} & =-\beta \langle k\rangle s(t)i(t)-\sigma s(t)i(t), \end{array}$$

(2) $$\begin{array}{cc} \frac{de(t)}{dt} & =\beta \langle k\rangle s(t)i(t)-\alpha e(t), \end{array}$$

(3) $$\begin{array}{cc} \frac{di(t)}{dt} & =\alpha e(t)-\mu i(t)+\sigma s(t)i(t), \end{array}$$

(4) $$\begin{array}{cc} \frac{dr(t)}{dt} & =\mu i(t). \end{array}$$

At any time *t*, the four compartments always satisfy s(t)+e(t)+i(t)+r(t)=1. In the initial stage, most nodes are ignorant of the information, and we have s(0)≃1, and we assume that there are few infected individuals in the system, so that i(0)≃0. Similarly, very few nodes are exposed or recovered, giving e(0)≃0 and r(0)≃0. Ignoring $i^{2}$ terms, we have

(5) $$\begin{array}{cc} \frac{de(t)}{dt} & =\beta \langle k\rangle i(t)-\alpha e(t), \end{array}$$

(6) $$\begin{array}{cc} \frac{di(t)}{dt} & =\alpha e(t)-\mu i(t)+\sigma i(t). \end{array}$$

The system in the initial stage of spreading is primarily governed by the exposed and infected nodes. This is a dynamical system of ordinary differential equations, which can be written in matrix form

(7) $$\begin{array}{c} \frac{d}{dt}\left[\begin{array}{c} e(t)\\ i(t) \end{array}\right]=A\left[\begin{array}{c} e(t)\\ i(t) \end{array}\right]=\left[\begin{array}{cc} -\alpha & \beta \langle k\rangle \\ \alpha & \sigma -\mu \end{array}\right]\left[\begin{array}{c} e(t)\\ i(t) \end{array}\right]. \end{array}$$

To solve the differential equations, we calculate the eigenvalues of matrix *A*, which we denote as $\lambda_{1,2}$. Specifically, the eigenvalues are

(8) $$\begin{array}{cc} \lambda_{1} & =\frac{-\left(\alpha -\sigma +\mu \right)+\sqrt{{\left(\alpha -\sigma +\mu \right)}^{2}+4\alpha \left(\sigma -\mu +\beta \langle k\rangle \right)}}{2}, \end{array}$$

(9) $$\begin{array}{cc} \lambda_{2} & =\frac{-\left(\alpha -\sigma +\mu \right)-\sqrt{{\left(\alpha -\sigma +\mu \right)}^{2}+4\alpha \left(\sigma -\mu +\beta \langle k\rangle \right)}}{2}. \end{array}$$

The analytical solutions for e(t) and i(t) in the initial spreading stage can be expressed as

(10) $$\begin{array}{cc} e(t) & =C_{1}\beta \langle k\rangle e^{\lambda_{1}t}+C_{2}\beta \langle k\rangle e^{\lambda_{2}t}, \end{array}$$

(11) $$\begin{array}{cc} i(t) & =C_{1}\left(\alpha +\lambda_{1}\right)e^{\lambda_{1}t}+C_{2}\left(\alpha +\lambda_{2}\right)e^{\lambda_{2}t}, \end{array}$$

with $C_{1}$ and $C_{2}$ being coefficients that can be determined by the initial conditions of e(0) and i(0). As $\lambda_{2}<0$, the second term of i(t)$e^{\lambda_{2}t}\to 0$ as *t* increases. Therefore, the dynamic of i(t) is primarily controlled by $i\left(t\right)≃C_{1}\left(\alpha +\lambda_{1}\right)e^{\lambda_{1}t}$, which suggests that at the initial stage of information diffusion, the spreading process follows an exponential growth dynamic. We simplify $\lambda_{1}$ as

(12) $$\begin{array}{c} \begin{array}{cc} \lambda_{1} & =\frac{-\left(\alpha -\sigma +\mu \right)+\sqrt{{\left(\alpha -\sigma +\mu \right)}^{2}+4\alpha \left(\sigma -\mu +\beta \langle k\rangle \right)}}{2}\\ \; & =\frac{2\alpha (\sigma -\mu +\beta \langle k\rangle )}{\left(\alpha -\sigma +\mu \right)+\sqrt{{\left(\alpha -\sigma +\mu \right)}^{2}+4\alpha \left(\sigma -\mu +\beta \langle k\rangle \right)}}\\ \; & ≃\frac{\alpha (\sigma -\mu +\beta \langle k\rangle )}{\alpha -\sigma +\mu }, \end{array} \end{array}$$

where we have assumed that α≪μ,σ, and we have neglected the term α(σ−μ+β〈k〉) in the equation. This is a simplifying assumption to obtain a simple form of the square root expression in the denominator. In real-world scenarios, this applies to the cases when the information is not super attractive to individuals, and they are slow to decide to spread the information and would quickly forget about it. As most information or knowledge will not become viral, this scenario may be very common in real-world diffusion events. In case of a viral diffusion, when this assumption is not satisfied, and μ<σ+β〈k〉, this expression gives an upper bound estimate of the exact value of $\lambda_{1}$.

The typical outbreak time scale Γ can be given as

(13) $$\begin{array}{c} \begin{array}{cc} \Gamma =\lambda_{1}^{-1} & =\frac{\alpha -\sigma +\mu }{\alpha (\sigma -\mu +\beta \langle k\rangle )}\\ \; & =\frac{1+(\mu -\sigma )/\alpha }{\sigma +\beta \langle k\rangle -\mu }. \end{array} \end{array}$$

To obtain a more concise form of time scale Γ, we assume that 〈k〉≫1, yielding

(14) $$\begin{array}{c} \Gamma ≃\frac{1}{\langle k\rangle }\frac{1+(\mu -\sigma )/\alpha }{\beta }. \end{array}$$

This suggests that the outbreak time scale Γ reduces when the network connectivity 〈k〉≫1 increases. Increasing the infection rate β, exposure rate α, and the rate of independent spreaders σ can also reduce the outbreak time scale, making the information diffusion faster in the initial stage.

We present analyses of the time scale of the SEIR model with independent spreaders in homogeneous networks. We ran the simulations on a Small World network with *N* = 20,000 nodes and mean degree 〈k〉=20 [36]. We then defined the time scale $\Gamma^{*}$ as the average minimum time when the system has 5% recovered nodes

(15) $$\begin{array}{c} \Gamma^{*}=\arg\;\min\left\{t|r\left(t\right)\geq 5\%\right\}. \end{array}$$

**Figure 2:**
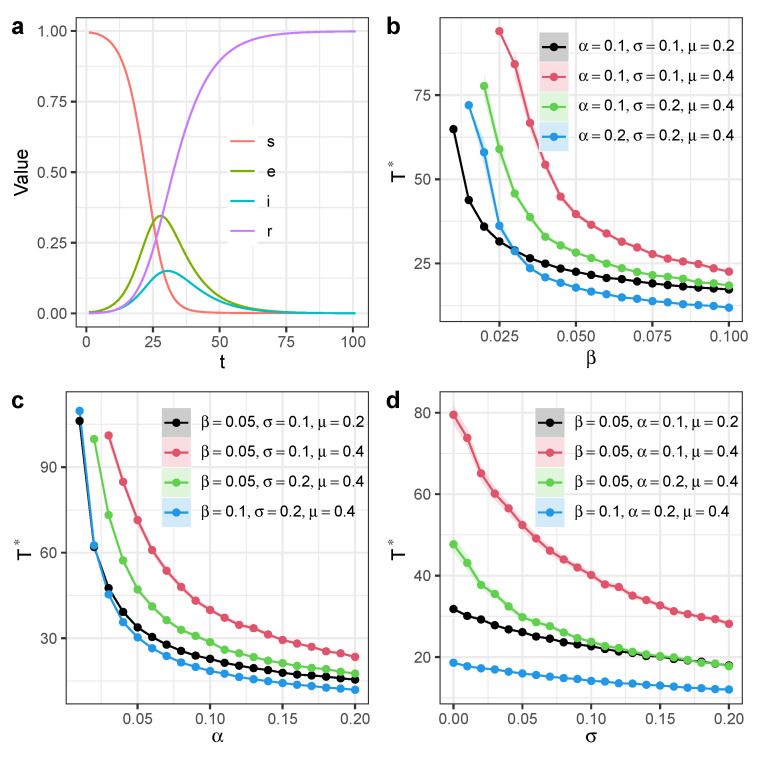
Time scale of SEIR model with independent spreaders in homogeneous networks. We ran simulations on a Small World network with *N* = 20,000 nodes and mean degree 〈k〉=20. (**a**) Time dynamics of information diffusion process with β=0.05,α=0.1,σ=0.1,μ=0.2. (**b**) Time scale $\Gamma^{*}$ as a function of infection rate β. (**c**) Time scale $\Gamma^{*}$ as a function of exposed-to-infection rate α. (**d**) Time scale $\Gamma^{*}$ as a function of activeness of independent spreaders σ. Each dot denotes the average value from 100 simulations. Different colors represent different model parameter configurations. Shaded areas represent 95% confidence intervals.

We show the time scale $\Gamma^{*}$ as a function of infection rate β and find that increasing β can reduce $\Gamma^{*}$ (Figure 2b). Similarly, increasing the exposed-to-infection rate α can also reduce $\Gamma^{*}$ (Figure 2c). The participation of independent spreaders can also reduce the time scale $\Gamma^{*}$, and the activeness of independent spreaders σ has a linear relation with $\Gamma^{*}$ when σ becomes large (Figure 2d).

We next examined the long-term limit of information diffusion outbreaks in homogeneous networks. From Equation (1), we obtain the analytical form of s(t) as

(16) $$\begin{array}{cc} s(t) & =e^{-\left(\beta \langle k\rangle +\sigma \right)\int_{0}^{t}i\left(\tau \right)d\tau } \end{array}$$

(17) $$\begin{array}{cc} r(t) & =\mu \int_{0}^{t}i\left(\tau \right)d\tau . \end{array}$$

At the long-term limits, we have t→∞, and the system reaches equilibrium, suggesting that

(18) $$\begin{array}{c} \frac{ds(t)}{dt}{|}_{t\to \infty }=\frac{de(t)}{dt}{|}_{t\to \infty }=\frac{di(t)}{dt}{|}_{t\to \infty }=\frac{dr(t)}{dt}{|}_{t\to \infty }=0. \end{array}$$

From Equations (1)–(4), we obtain that $e_{\infty }=0$, and $i_{\infty }=0$; thus, $s_{\infty }+r_{\infty }=1$. From Equation (17), we have $s_{\infty }=e^{-\frac{\beta \langle k\rangle +\sigma }{\mu }r_{\infty }}$, and it is straightforward to obtain the self-consistent equation

(19) $$\begin{array}{c} r_{\infty }=1-e^{-\frac{\beta \langle k\rangle +\sigma }{\mu }r_{\infty }}. \end{array}$$

This equation always has a trivial solution of $r_{\infty }^{*}=0$. We derive the condition under which it has a non-trivial solution where $0<r_{\infty }^{*}\leq 1$, only when

(20) $$\begin{array}{c} \frac{d}{dr_{\infty }}(1-e^{-\frac{\beta \langle k\rangle +\sigma }{\mu }r_{\infty }}){|}_{r_{\infty }=0}\geq 1. \end{array}$$

Solving this inequality leads to the definition of a crucial epidemiological concept, the information diffusion threshold, which reads

(21) $$\begin{array}{c} \frac{\beta }{\mu -\sigma }\geq \frac{1}{\langle k\rangle }. \end{array}$$

If the spreading rate is not large enough, e.g., when β<1/(〈k〉(μ−σ)), the information diffusion will only affect a finite proportion of the population and will extinguish in a finite time. With better network connectivity as 〈k〉 increases, a larger diffusion rate β or a larger rate of independent spreaders σ, the diffusion threshold will diminish, making the information or knowledge easier to spread to a significant proportion of the population.

**Figure 3:**
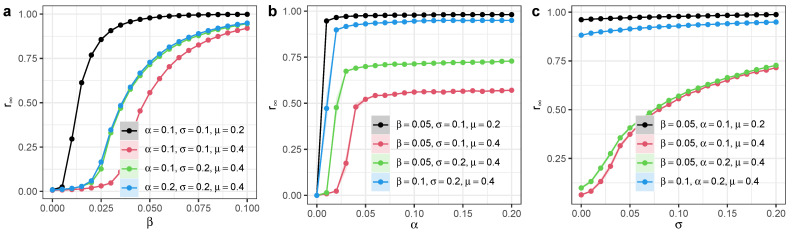
Long-term steady state of SEIR model with independent spreaders in homogeneous networks. We ran the simulation on a Small World network with *N* = 20,000 nodes and mean degree 〈k〉=20. (**a**) Long-term recovered nodes $r_{\infty }$ as a function of infection rate β. (**b**) Long-term recovered nodes $r_{\infty }$ as a function of exposed-to-infection rate α. (**c**) Long-term recovered nodes $r_{\infty }$ as a function of activeness of independent spreaders σ. Each dot denotes the average value from 100 simulations. Different colors represent different model parameter configurations. Shaded areas represent 95% confidence intervals.

We ran simulations on a Small World network with *N* = 20,000 nodes and mean degree 〈k〉=20. We found that the long-term recovered nodes $r_{\infty }$ increase as the infection rate β increases, especially when β becomes larger (Figure 3a). Yet, when α is positive, $r_{\infty }$ does not change significantly as α increases (Figure 3b). The participation of independent spreaders can substantially increase the fraction of the recovered nodes $r_{\infty }$, and the activeness of independent spreaders σ has a linear relation with $r_{\infty }$ (Figure 3c). These results demonstrate the importance of independent spreaders in facilitating information or knowledge diffusion processes in homogeneous complex networks.

### 2.2. Information Diffusion on Heterogeneous Networks

Many real-world complex social networks have varying degree distributions, and the homogeneous mixing hypothesis used in the previous section applies to only a very small fraction of networks. In many empirical networks, such as the internet, social network platforms, and disease contact networks, the degree distribution can be heavy-tailed, and some nodes with extremely high connectivity can act as hubs or “super-spreaders”, which have broad influence on the spreading dynamics in the networks. Recent studies have emphasized the role of heterogeneity by showing that disease or information transmissibility is a positive and increasing function of the deviation of connectivity patterns. In these scenarios, the homogeneous assumption that every node has approximately the same connectivity does not fit to the topology structure of the underlying network, and we need to account for the variation in the degree distribution of nodes.

In this section, we examine the SEIR information diffusion dynamics with the participation of independent spreaders in heterogeneous networks. We adopted a degree block approximation that assumes that all nodes with the same degree are statistically equivalent. This assumption allowed us to group nodes in the same degree class *k* and yielded a representation of the system by quantities such as the density of susceptible nodes, exposed nodes, infected nodes, and recovered nodes [37]. We specify the SEIR information diffusion model on heterogeneous complex networks in the following equations:

(22) $$\begin{array}{c} s_{k}=\frac{S_{k}}{N_{k}};\;e_{k}=\frac{E_{k}}{N_{k}};\;i_{k}=\frac{I_{k}}{N_{k}};\;r_{k}=\frac{R_{k}}{N_{k}}, \end{array}$$

where $N_{k}$ is the number of nodes with degree *k*, and $S_{k}$, $E_{k}$, $I_{k}$, and $R_{k}$ are the number of susceptible, exposed, infected, and recovered nodes in that class, respectively. The global average density of these states are given by

(23) $$\begin{array}{c} s\left(t\right)=\sum_{k}P\left(k\right)s_{k}\left(t\right);\;\;e\left(t\right)=\sum_{k}P\left(k\right)e_{k}\left(t\right);\;\;i\left(t\right)=\sum_{k}P\left(k\right)i_{k}\left(t\right);\;\;r\left(t\right)=\sum_{k}P\left(k\right)r_{k}\left(t\right), \end{array}$$

where P(k) is the proportion of nodes with degree *k* in the network. We assumed that the heterogeneous network has no degree correlations, which suggests that the probability that an edge departing from a node of degree *k* arrives at another node of degree $k^{\prime }$ is independent of *k*.

The no degree correlation assumption is to simplify the differential equations of the compartment epidemic models and obtain analytical derivations inthe critical diffusion time and outbreak threshold, which we show in the following analysis. For networks with degree correlations, the network topology is more complicated. Assortative networks may facilitate faster information spreading among hubs but could isolate peripheral nodes, while disassortative networks may enable efficient information transfer from hubs to low-degree nodes, promoting broader dissemination.

Under this no-degree correlation assumption, the conditional probability does not depend on the originating node, and it is possible to show that $P\left(k^{\prime }|k\right)=k^{\prime }P\left(k^{\prime }\right)/\langle k\rangle$. Considering that at least one of the edges of the infected node links to another infected node from which the information has been diffused, this yields

(24) $$\begin{array}{c} \Theta_{k}\left(t\right)=\Theta \left(t\right)=\frac{\sum_{k^{\prime }}\left(k^{\prime }-1\right)P\left(k^{\prime }\right)i_{k^{\prime }}\left(t\right)}{\langle k\rangle }. \end{array}$$

For each class of degree *k*, the evolution equations of information diffusion dynamics are

(25) $$\begin{array}{cc} \frac{ds_{k}\left(t\right)}{dt} & =-\beta ks_{k}\left(t\right)\Theta \left(t\right)-\sigma s_{k}\left(t\right)\Theta \left(t\right), \end{array}$$

(26) $$\begin{array}{cc} \frac{de_{k}\left(t\right)}{dt} & =\beta ks_{k}\left(t\right)\Theta \left(t\right)-\alpha e_{k}\left(t\right), \end{array}$$

(27) $$\begin{array}{cc} \frac{di_{k}\left(t\right)}{dt} & =\alpha e_{k}\left(t\right)-\mu i_{k}\left(t\right)+\sigma s_{k}\left(t\right)\Theta \left(t\right), \end{array}$$

(28) $$\begin{array}{cc} \frac{dr_{k}\left(t\right)}{dt} & =\mu i_{k}\left(t\right). \end{array}$$

In the initial information diffusion stages, we obtain the evolution equations for Θ(t), by neglecting terms of the order $O(i^{2})$. The equations are

(29) $$\begin{array}{cc} \frac{de_{k}\left(t\right)}{dt} & =\beta k\Theta \left(t\right)-\alpha e_{k}\left(t\right), \end{array}$$

(30) $$\begin{array}{cc} \frac{di_{k}\left(t\right)}{dt} & =\alpha e_{k}\left(t\right)-\mu i_{k}\left(t\right)+\sigma \Theta \left(t\right). \end{array}$$

To simplify the discussion of Θ(t), we define

(31) $$\begin{array}{c} E\left(t\right)\equiv \frac{1}{\langle k\rangle }\sum_{k^{\prime }}\left(k^{\prime }-1\right)P\left(k^{\prime }\right)e_{k}\left(t\right) \end{array}$$

to express the average rate of exposed nodes in the network. By multiplying the term $\frac{1}{\langle k\rangle }\sum_{k^{\prime }}\left(k^{\prime }-1\right)P\left(k^{\prime }\right)$ on both sides of Equation (30) and summing up as a function of degree *k*, we obtain the expressions of E(t) and Θ(t) as

(32) $$\begin{array}{cc} \frac{dE(t)}{dt} & =\beta \frac{\left(\langle k^{2}\rangle -\langle k\rangle \right)}{\langle k\rangle }\Theta \left(t\right)-\alpha E\left(t\right), \end{array}$$

(33) $$\begin{array}{cc} \frac{d\Theta (t)}{dt} & =\alpha E\left(t\right)-\mu \Theta \left(t\right)+\left(1-\frac{1}{\langle k\rangle }\right)\sigma \Theta \left(t\right). \end{array}$$

By solving the differential equations, we yield analytical forms of E(t) and Θ(t). Analogous to the analyses in the homogeneous networks, we obtain the typical time scale of information diffusion outbreaks as

(34) $$\begin{array}{c} \begin{array}{c} \Gamma =\frac{1+(\mu -\frac{\langle k\rangle -1}{\langle k\rangle }\sigma )/\alpha }{\frac{\langle k\rangle -1}{\langle k\rangle }\sigma +\frac{\langle k^{2}\rangle -\langle k\rangle }{\langle k\rangle }\beta -\mu }. \end{array} \end{array}$$

This expression suggests that the time scale of the information diffusion outbreak in heterogeneous networks is dominated by the term $\langle k^{2}\rangle$.

(35) $$\begin{array}{c} \Gamma ≃\frac{\langle k\rangle }{\langle k^{2}\rangle }\frac{1+(\mu -\sigma )/\alpha }{\beta }. \end{array}$$

The outbreak time scale of the information diffusion on heterogeneous networks is governed by structural properties $\langle k\rangle /\langle k^{2}\rangle$. Similar to the case in homogeneous networks, increasing the infection rate β, exposure rate α, and the rate of independent spreaders σ can also reduce the outbreak time scale, making the information diffusion faster in the initial stage.

**Figure 4:**
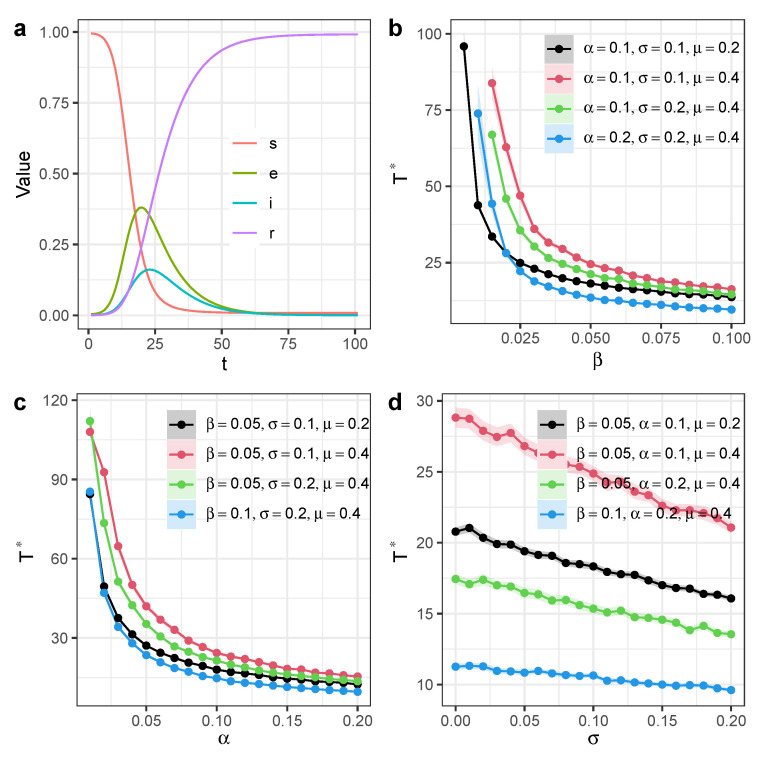
Time scale of SEIR model with independent spreaders in heterogeneous networks. We ran simulations on a Scale-Free network with *N* = 20,000 nodes and mean degree 〈k〉=20. (**a**) Time dynamics of information diffusion process with β=0.05,α=0.1,σ=0.1,μ=0.2. (**b**) Time scale $\Gamma^{*}$ as a function of infection rate β. (**c**) Time scale $\Gamma^{*}$ as a function of exposed-to-infection rate α. (**d**) Time scale $\Gamma^{*}$ as a function of activeness of independent spreaders σ. Each dot denotes the average value from 100 simulations. Different colors represent different model parameter configurations. Shaded areas represent 95% confidence intervals.

Running extensive computational simulation on a Scale-Free network with *N* = 20,000 nodes and mean degree 〈k〉=20, we showed the time scale $\Gamma^{*}$ as a function of infection rate β and determined that increasing β can reduce $\Gamma^{*}$ (Figure 4b) [38]. Similarly, increasing the exposed-to-infection rate α can also reduce $\Gamma^{*}$ (Figure 4c). The participation of independent spreaders can also reduce the time scale $\Gamma^{*}$, and the activeness of independent spreaders σ has a linear relation with $\Gamma^{*}$ when σ becomes large (Figure 4d).

Next, we examined the long-term steady state of the information diffusion process in heterogeneous networks. From Equations (25) and (28), we yield the expressions

(36) $$\begin{array}{c} s_{k}\left(t\right)=e^{-(\beta k+\sigma )\phi (t)};\;r_{k}\left(t\right)=\mu \int_{0}^{t}i_{k}\left(\tau \right)d\tau , \end{array}$$

where the auxiliary function ϕ(t) is defined as

(37) $$\begin{array}{c} \phi \left(t\right)\equiv \int_{0}^{t}\Theta \left(\tau \right)d\tau =\frac{1}{\langle k\rangle \mu }\sum_{k^{\prime }}\left(k^{\prime }-1\right)P\left(k^{\prime }\right)r_{k^{\prime }}\left(t\right). \end{array}$$

To yield a closed relation for the total density of infected nodes, it is more convenient to focus on the time evolution of ϕ(t). We compute its time derivative

(38) $$\begin{array}{c} \begin{array}{cc} \frac{d\phi (t)}{dt} & =\frac{1}{\langle k\rangle }\sum_{k}\left(k-1\right)P\left(k\right)i_{k}\left(t\right)\\ \; & =\frac{1}{\langle k\rangle }\sum_{k}\left(k-1\right)P\left(k\right)\left[1-r_{k}\left(t\right)-s_{k}\left(t\right)-e_{k}\left(t\right)\right]\\ \; & =1-\frac{1}{\langle k\rangle }-\mu \phi \left(t\right)-\frac{1}{\langle k\rangle }\sum_{k}\left(k-1\right)P\left(k\right)e^{-(\beta k+\sigma )\phi (t)}-E\left(t\right), \end{array} \end{array}$$

where we have used the time dependence of $s_{k}\left(t\right)$ derived in Equations (36) and (37). For a general distribution P(k), Equation (38) cannot be solved in a closed form, but it is possible to obtain useful information on the infinite time.

When t→∞,

(39) $$\begin{array}{c} \frac{ds_{k}\left(t\right)}{dt}{|}_{t\to \infty }=\frac{de_{k}\left(t\right)}{dt}{|}_{t\to \infty }=\frac{di_{k}\left(t\right)}{dt}{|}_{t\to \infty }=\frac{dr_{k}\left(t\right)}{dt}{|}_{t\to \infty }=0. \end{array}$$

This gives $i_{k}\left(\infty \right)=0$, $e_{k}\left(\infty \right)=0$, and E(∞)=0. The total information prevalence $r_{\infty }=\sum_{k}P\left(k\right)r_{k}\left(\infty \right)$ is yielded as a function of $\phi_{\infty }=\lim_{t\to \infty }\phi \left(t\right)$

(40) $$\begin{array}{c} r_{\infty }=\sum_{k}P\left(k\right)\left(1-e^{-(\beta k+\sigma )\phi (t)}\right), \end{array}$$

where we have used $s_{\infty }+r_{\infty }=1$. As $\lim_{t\to \infty }d\phi \left(t\right)/dt=0$, we obtain from Equation (38) the self-consistent equation for $\phi_{\infty }$:

(41) $$\begin{array}{c} \mu \phi_{\infty }=1-\frac{1}{\langle k\rangle }-\frac{1}{\langle k\rangle }\sum_{k}\left(k-1\right)P\left(k\right)e^{-\left(\beta k+\sigma \right)\phi_{\infty }}. \end{array}$$

The equation always has a trivial solution of $\phi_{\infty }=0$. The non-zero $\phi_{\infty }$ solution, corresponding to the finite prevalence at the long-term limit, exists only if

(42) $$\begin{array}{c} \frac{d}{d\phi_{\infty }}(1-\frac{1}{\langle k\rangle }-\frac{1}{\langle k\rangle }\sum_{k}\left(k-1\right)P\left(k\right)e^{-\left(\beta k+\sigma \right)\phi_{\infty }}){|}_{\phi_{\infty }=0}\geq \mu . \end{array}$$

This condition is equivalent to

(43) $$\begin{array}{c} \frac{(\langle k^{2}\rangle -\langle k\rangle )\beta }{\langle k\rangle \mu -(\langle k\rangle -1)\sigma }\geq 1. \end{array}$$

If 〈k〉≫1, we obtain a more concise form of prevalence condition as

(44) $$\begin{array}{c} \frac{\beta }{\mu -\sigma }\geq \frac{\langle k\rangle }{\langle k^{2}\rangle -\langle k\rangle }. \end{array}$$

The diffusion prevalence condition implies that there is a null information diffusion threshold in heavy-tailed networks such that $\langle k^{2}\rangle \to \infty$ in the limit of networks with infinite size. While this is not the case in real-world networks with finite sizes, larger heterogeneity levels lead to smaller information diffusion thresholds. The parameter $\kappa =\langle k^{2}\rangle /\langle k\rangle$ that defines heterogeneity in the connectivity pattern determines the social processes occurring on this network. Moreover, the generating probability of independent spreaders σ reduces the diffusion threshold when other diffusion and network structural parameters remain constant, which demonstrates the important role of independent spreaders in facilitating information and knowledge sharing in social networks.

**Figure 5:**
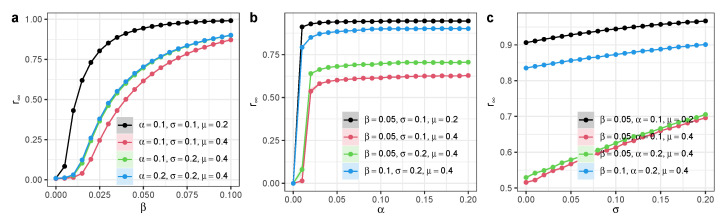
Long-term steady state of SEIR model with independent spreaders in heterogeneous networks. We ran the simulation on a Scale-Free network with *N* = 20,000 nodes and mean degree 〈k〉=20. (**a**) Long-term recovered nodes $r_{\infty }$ as a function of infection rate β. (**b**) Long-term recovered nodes $r_{\infty }$ as a function of exposed-to-infection rate α. (**c**) Long-term recovered nodes $r_{\infty }$ as a function of activeness of independent spreaders σ. Each dot denotes the average value from 100 simulations. Different colors represent different model parameter configurations. Shaded areas represent 95% confidence intervals.

We ran simulations on a Scale-Free network with *N* = 20,000 nodes and mean degree 〈k〉=20. We determined that the long-term recovered nodes $r_{\infty }$ increased as the infection rate β increased, especially when β became larger (Figure 5a). Yet, when α was positive, $r_{\infty }$ did not change significantly as α increased (Figure 5b). The participation of independent spreaders can substantially increase the fraction of recovered nodes $r_{\infty }$, and the activeness of independent spreaders σ has a linear relation with $r_{\infty }$ (Figure 5c). These results demonstrate the importance of independent spreaders in facilitating information or knowledge diffusion processes in heterogeneous complex networks.

### 2.3. Information Diffusion on Real World Social Networks

We simulated our model on two large scale real-world social networks. One was the artist network of blue verified Facebook page networks, where nodes represented the pages, and edges were mutual likes among them [39]. The network had 50,515 nodes, and 819,306 edges, with mean degree 〈k〉=32.44. The network was composed of one giant component, and all nodes were connected with link paths.

We also constructed the collaboration network of physicists over six decades from 1950 to 2016, using the publication data of papers published by the American Physical Society (APS) [40]. The original APS collaboration network contained 219,640 nodes and 1,185,427 edges with mean degree 〈k〉=10.79. However, the raw network composed many small subgraphs not connected to other parts of the network, and the information diffusion process could not reach these isolated network components. We trimmed the network and extracted the largest connecting component, which had 201,660 nodes and 1,167,200 edges with mean degree 〈k〉=11.58.

With the APS physics publication data, we also extracted the collaborative relations among research institutions and universities to build the network at the level of institutions [41]. The data included institutions and universities from major countries, including the United States of America, the European Union, and China. The network had *N* = 11,478 nodes, with each node representing a research institution or university, and 256,934 edges, with mean degree 〈k〉=44.77. The connectivity of the institution collaboration network was better than the researcher collaboration network, suggesting that institutions or universities have more interactions than individual researchers.

**Figure 6:**
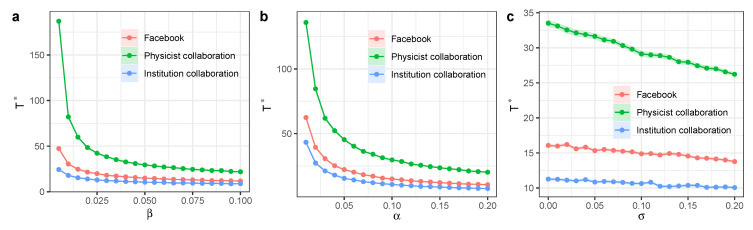
Time scale of SEIR model with independent spreaders in real-world social networks. We ran the simulation on a Facebook artists’ network with 50,515 nodes with mean degree 〈k〉=32.44, the APS physicist collaboration network with *N* = 201,660 nodes and mean degree 〈k〉=11.58, and the physics institutional collaboration network with *N* = 11,478 nodes and mean degree 〈k〉=44.77. (**a**) Time scale $\Gamma^{*}$ as a function of infection rate β. Other parameters are α=0.1,σ=0.1, and μ=0.2. (**b**) Time scale $\Gamma^{*}$ as a function of exposed-to-infection rate α. Other parameters are β=0.05, σ=0.1, and μ=0.2. (**c**) Time scale $\Gamma^{*}$ as a function of activeness of independent spreaders σ. Other parameters are β=0.05,α=0.1,andμ=0.2. Each dot denotes the average value from 100 simulations. Different colors represent different model parameter configurations. Shaded areas represent 95% confidence intervals.

We ran extensive simulations on the Facebook artist network, the APS collaboration network of physicists, and the physics institutional collaboration network. We showed the time scale $\Gamma^{*}$ as a function of infection rate β and determined that increasing β can reduce $\Gamma^{*}$ (Figure 6a). Similarly, increasing the exposed-to-infection rate α can also reduce $\Gamma^{*}$ (Figure 6b). The participation of independent spreaders can also reduce the time scale $\Gamma^{*}$, and the activeness of independent spreaders σ has a linear relation with $\Gamma^{*}$ when σ becomes large (Figure 6c).

**Figure 7:**
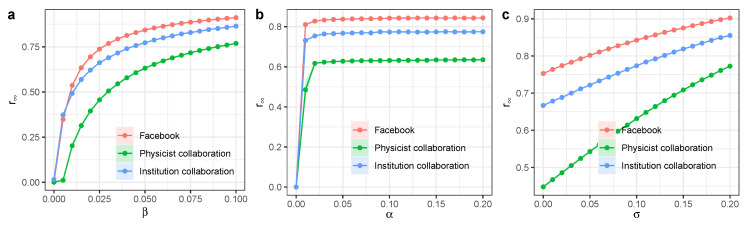
Long-term steady state of SEIR model with independent spreaders in real-world social networks. We ran the simulation on a Facebook artists’ network with 50,515 nodes with mean degree 〈k〉=32.44, the APS physicist collaboration network with *N* = 201,660 nodes and mean degree 〈k〉=11.58, and the physics institutional collaboration network with *N* = 11,478 nodes and mean degree 〈k〉=44.77. (**a**) Long-term recovered nodes $r_{\infty }$ as a function of infection rate β. Other parameters are α=0.1,σ=0.1, and μ=0.2. (**b**) Long-term recovered nodes $r_{\infty }$ as a function of exposed-to-infection rate α. Other parameters are β=0.05,σ=0.1, and μ=0.2. (**c**) Long-term recovered nodes $r_{\infty }$ as a function of activeness of independent spreaders σ. Other parameters are β=0.05,α=0.1, and μ=0.2. Each dot denotes the average value from 100 simulations. Different colors represent different model parameter configurations. Shaded areas represent 95% confidence intervals.

Summarizing the simulation results from these social networks, we determined that the long-term recovered nodes $r_{\infty }$ increased as the infection rate β increased, especially when β became larger (Figure 7a). Yet, when α was positive, $r_{\infty }$ did not change significantly as α increased (Figure 7b). The participation of independent spreaders can substantially increase the fraction of recovered nodes $r_{\infty }$, and the activeness of independent spreaders σ has a linear relation with $r_{\infty }$ (Figure 7c). These results demonstrate the importance of independent spreaders in facilitating information or knowledge diffusion processes in real-world social networks of individuals or institutions.

## 3. Comparative Analyses

To compare with other models, we provide benchmark comparisons of the independent spreader (IS) model with a range of other epidemic models. The first one was the baseline SEIR model without the independent spreaders. Then, we used the SEIR model with external seeding, which is the exogenous injection of infected nodes into the system. This process is often referred to as broadcasting on social networks [33]. The external seeding rate is defined as θ. Next, we introduced the SEIR model with hybrid diffusion combining endogenous and exogenous spreading [30]. In the hybrid diffusion process, we used external seeding as the exogenous spreading and followed Ref. [34] to define the endogenous function. The endogenous infection rate function is defined as $\beta \left(t\right)=\beta_{0}+d\phi \left(t\right)$, where *d* is the triggering rate of boosting the infection rate, and we used a uniform distribution for ϕ(t).

**Figure 8:**
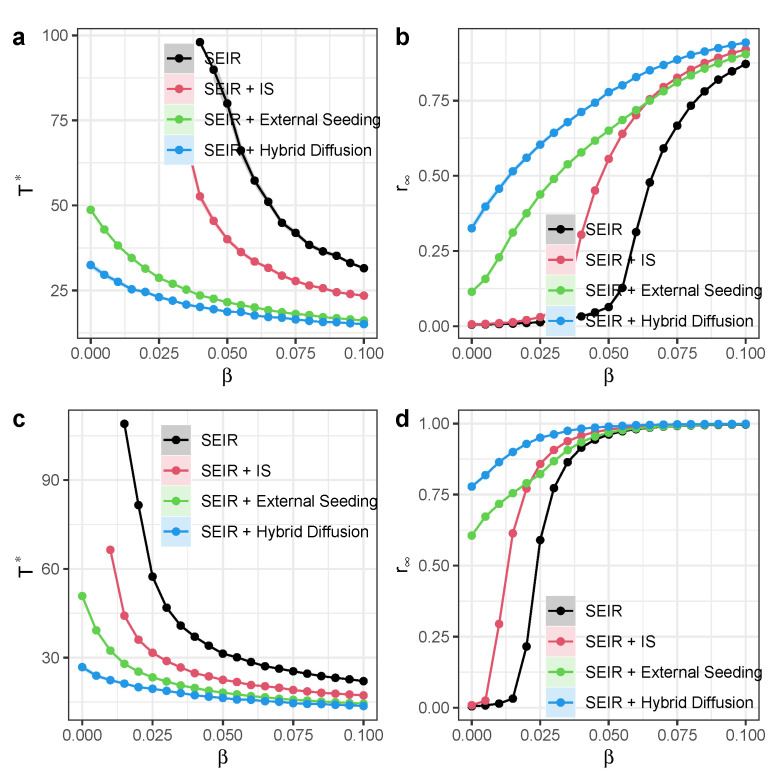
Experimental performance comparison between the independent spreaders model with other models. (**a**) Time scale $\Gamma^{*}$ as a function of infection rate β, with μ=0.4. (**b**) Long-term recovered nodes $r_{\infty }$ as a function of exposed-to-infection rate β, with μ=0.4. (**c**) Time scale $\Gamma^{*}$ as a function of infection rate β, with μ=0.2. (**d**) Long-term recovered nodes $r_{\infty }$ as a function of exposed-to-infection rate β, with μ=0.2. Other parameters are α=0.1, external seeding rate θ=0.001, and ϕ(t) is a uniform distribution (0.1,0.3) with triggering rate d=0.2. Each dot denotes the average value from 100 simulations. Different colors represent different models. Shaded areas represent 95% confidence intervals.

We ran the models on the Small-World networks to compare the benchmarks between the proposed model and other models, such as external seeding and hybrid diffusion (Figure 8) [31,32]. We systematically analyzed the performance metrics across different models, which included the outbreak speed, final spread size, sensitivity to parameter variations, and diffusion thresholds. It seems that the SEIR and hybrid diffusion model had the fastest outbreak speed and the largest final spread size among these models, while the basic SEIR model had the slowest outbreak speed and the smallest final spread size. When we changed μ, all the models’ performance changed. The proposed independent spreader model had smaller diffusion thresholds than the basic SEIR model, while both the SEIR and external seeding and the SEIR and hybrid diffusion models had no diffusion thresholds.

**Figure 9:**
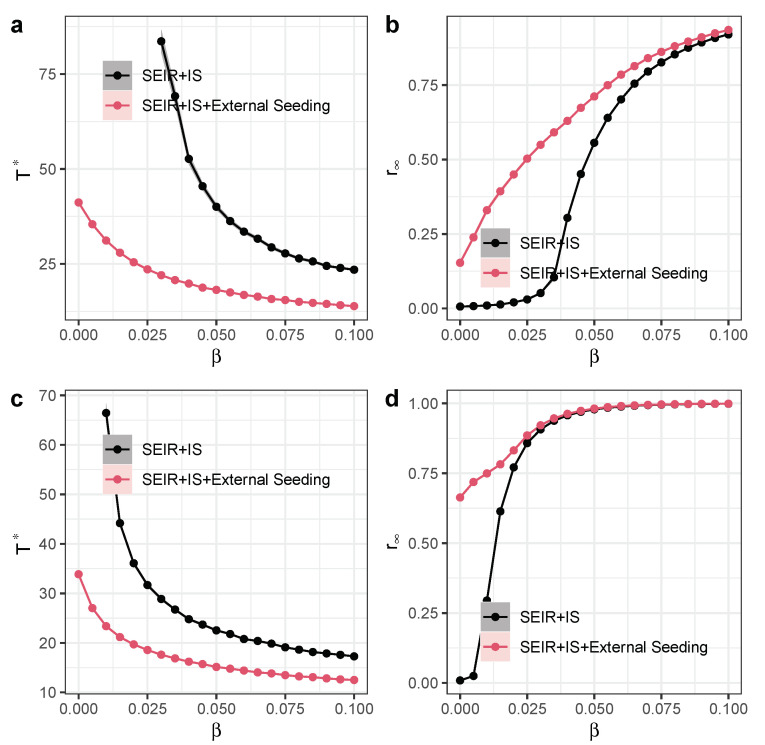
Quantification of exogenous influence in the diffusion model. (**a**) Time scale $\Gamma^{*}$ as a function of infection rate β, with μ=0.4. (**b**) Long-term recovered nodes $r_{\infty }$ as a function of exposed-to-infection rate β, with μ=0.4. (**c**) Time scale $\Gamma^{*}$ as a function of infection rate β, with μ=0.2. (**d**) Long-term recovered nodes $r_{\infty }$ as a function of exposed-to-infection rate β, with μ=0.2. Other parameters are α=0.1, external seeding rate θ=0.001, and σ=0.1. Each dot denotes the average value from 100 simulations. Different colors represent different models. Shaded areas represent 95% confidence intervals.

We then provide quantification of exogenous influence in the diffusion model. Many existing studies empirically measure exogenous influences using real-world data (e.g., external media events triggering infection surges). We used external seeding to emulate media events triggering infections within the focal network. Then, we examined how it interacted with or compared to other external effects, such as the external seeding process, which we showed in Figure 9. When independent spreaders come into play with external seeding in the diffusion process, the critical diffusion time decreases substantially, while the final spread size has increased considerably. The interaction can enhance the diffusion process, especially when the infection rate β is small.

**Figure 10:**
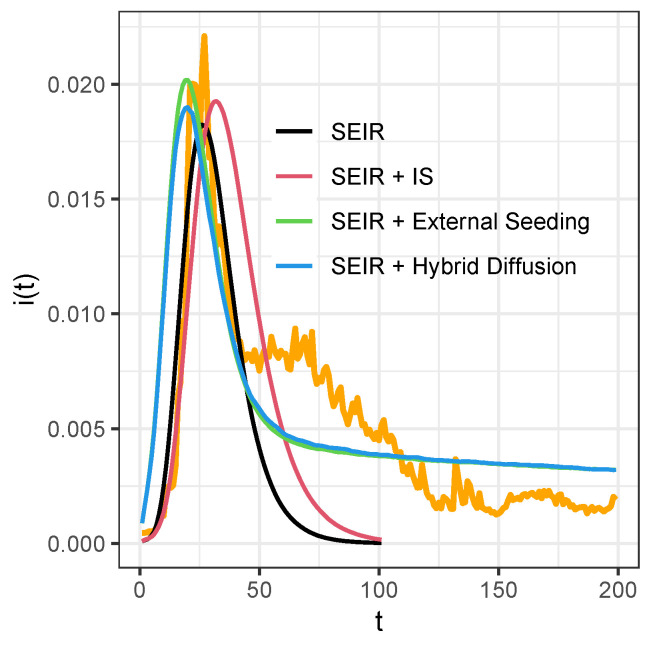
Real-world performance validation of the models with retweet data in Twitter. We show the fit-to-data evaluation of several models using empirical Twitter retweet data. The shown epidemic curves are from the models with good fit to data. Diffusion rates for different models are β=0.045 for SEIR model, β=0.03 for the SEIR and independent spreader model, β=0.035 for the SEIR and external seeding model, and β=0.015 for the SEIR and hybrid diffusion model. Other parameters are α=0.3, μ=0.2, external seeding rate θ=0.001, and σ=0.1. The orange curve represents the retweet data from Twitter. We ran 100 simulations for all models. Different colors represent different models.

We used real-world information diffusion data from the online network Twitter from Ref. [42]. The network contained 256,491 nodes and 328,132 edges, and the retweet data had 354,930 retweets. We then validated the performance of independent spreaders with other exogenous influence models in this practical setting. We showed the fit-to-data evaluation in Figure 10. In this practical scenario, we determined that the original SEIR model fit the central diffusion peak well, but it was not really good at the tail curve fitting. The independent spreaders model had really good fitting for the beginning, but not for the curve after the peak. The external seeding and hybrid diffusion models appeared to start the diffusion at an earlier stage, which suggests that the external seeding process in real-world scenarios may require additional parameters to limit its time scope.

## 4. Discussion

In this study, we examined the information and knowledge diffusion model with the SEIR process in complex networks. We provided analytical solutions for the diffusion outbreak time scale in the initial stage and the long-term outbreak size of the SEIR information diffusion dynamic systems in homogeneous networks, heterogeneous networks, and a set of empirical social networks. We also ran extensive computational simulations on synthetic homogeneous and heterogeneous networks, as well as two large-scale real-world social networks, one being the Facebook artist network and the other being the physicist collaboration network.

We determined that the participation of independent spreaders can substantially increase the fraction of recovered nodes. Independent spreaders can pass the information or knowledge to remote areas that are not well connected to other parts of the network, which facilitates the diffusion process of information or knowledge diffusion in complex social networks. Our theoretical and simulation analyses both demonstrate that the independent spreaders have a linear effect on both the critical time in the initial stage and the long-term steady state of the diffusion. The effects of independent spreaders persist across homogeneous and heterogeneous networks, as well as the real-world social networks of artists, researchers, and research institutions. We then compared the independent spreaders with other potential mechanisms that may also have exogenous influence, such as external seeding and hybrid diffusion in complex networks. We found that while they all enhanced the information diffusion in networks, the external seeding appeared to accelerate the diffusion speed since the very beginning, while independent spreaders still needed more time to make the information viral. All these mechanisms may have potential interactions in real-world scenarios, and exploring these potential usage and limitations of such mechanisms may be a potentially useful direction for future work.

These results demonstrate the importance of independent spreaders in facilitating information or knowledge diffusion processes in real-world social networks. Our model can help understand critical factors that affect information diffusion dynamics in online social network platforms and the innovation processes in universities and research institutions. Independent spreaders may play an important and unique role in the knowledge-sharing process, which may provide plausible measures for universities and research institutions to improve existing protocols and platforms to facilitate the spread of scientific ideas and innovations. The theoretical analysis and simulation findings may also have implications for future work that explores diffusion processes in complex social networks.

## Data Availability

The APS publication data can be accessed via https://journals.aps.org/datasets (accessed on 23 January 2025). The Facebook and Twitter data can be downloaded from https://snap.stanford.edu/ (accessed on 23 January 2025).
